# A new gestational diabetes mellitus model: hyperglycemia-induced eye malformation via inhibition of *Pax6* in the chick embryo

**DOI:** 10.1242/dmm.022012

**Published:** 2016-02-01

**Authors:** Shi-Jie Zhang, Yi-Fang Li, Rui-Rong Tan, Bun Tsoi, Wen-Shan Huang, Yi-Hua Huang, Xiao-Long Tang, Dan Hu, Nan Yao, Xuesong Yang, Hiroshi Kurihara, Qi Wang, Rong-Rong He

**Affiliations:** 1 Anti-stress and Health Research Center, College of Pharmacy, Jinan University, Guangzhou 510632, China; 2 Institute of Clinical Pharmacology, Guangzhou University of Chinese Medicine, Guangzhou 510006, China; 3 Guangdong Research Institute of Traditional Chinese Medicine Manufacturing Technology, Guangzhou 510095, China; 4 Division of Histology & Embryology, Key Laboratory for Regenerative Medicine of the Ministry of Education, Medical College, Jinan University, Guangzhou 510632, China

**Keywords:** Hyperglycemia, Eye malformation, *Pax6*, Oxidative stress, Chick embryo

## Abstract

Gestational diabetes mellitus (GDM) is one of the leading causes of fetal malformations. However, few models have been developed to study the underlying mechanisms of GDM-induced fetal eye malformation. In this study, a high concentration of glucose (0.2 mmol per egg) was injected into the air sac of chick embryos on embryo development day (EDD) 1 to develop a hyperglycemia model. Results showed that 47.3% of embryonic eye malformation happened on EDD 5. In this model, the key genes regulating eye development, *Pax6*, *Six3* and *Otx2*, were downregulated by hyperglycemia. Among these genes, the expression of *Pax6* was the most vulnerable to hyperglycemia, being suppressed by 70%. A reduction in *Pax6* gene expression induced eye malformation in chick embryos. However, increased expression of *Pax6* in chick embryos could rescue hyperglycemia-induced eye malformation. Hyperglycemia stimulated O-linked *N*-acetylglucosaminylation, which caused oxidative stress in chick embryos. Pax6 was found to be vulnerable to free radicals, but the antioxidant edaravone could restore Pax6 expression and reverse eye malformation. These results illustrated a successful establishment of a new chick embryo model to study the molecular mechanism of hyperglycemia-induced eye malformation. The suppression of the *Pax6* gene is probably mediated by oxidative stress and could be a crucial target for the therapy of GDM-induced embryonic eye malformation.

## Abstract (precis)

**Summary:** Hyperglycemia inhibited *Pax6* via oxidative stress and impaired eye development in the chick embryo, a new gestational diabetes mellitus model.

## INTRODUCTION

Gestational diabetes mellitus (GDM) is defined as ‘any degree of glucose intolerance with onset or first recognition during pregnancy’ (HAPO Study Cooperative Research Group et al., 2008). The worldwide incidence of GDM is increasing (Reece et al., 2009). Infants born to women with GDM are at an increased risk of adverse perinatal outcomes, such as congenital anomalies, macrosomia leading to birth trauma, hypoglycemia, respiratory distress and polycythemic jaundice (Burris and Camargo, 2014). Previous studies showed that maternal type I diabetes is associated with hypoplasia of the superior segmental optic nerve in offspring (Landau et al., 1998). Tariq et al. (2010) systematically investigated 2367 children (age 11.1-14.4 years) with complete detailed ocular examinations. Children from diabetic pregnancies had significantly thinner inner and outer macula lutea and lower macular volume compared with those from non-diabetic pregnancies. GDM-related eye malformations in children have become an important health problem, emphasizing the urgent need for resolution. However, there are few reports describing GDM-induced eye malformations.

To investigate GDM-induced eye malformations of the newborn, we proposed the development of a new chick embryo model of GDM. The chick embryo was chosen for the following four reasons: (i) the chick embryo is a classic developmental model, and the development of the embryonic eye has been extensively studied (Goodall et al., 2009); (ii) chick embryos have a rapid growth course (21 days in chicks compared with 9 months in humans; Martinsen, 2005); (iii) the chick embryo is separate from the maternal body, and thus will not be influenced by maternal metabolism; and (iv) the chick embryo is one of the simplest vertebrates, with well-characterized developmental stages. Thus, the effects of glucose on the development of embryos can be assessed easily. Glucose-induced malformations in embryos were related to the glucose dose and dependent on the developmental stage (Moley et al., 1996).

In this model, we exposed chick embryos to different concentrations of glucose on embryo development day (EDD) 1 to establish sustained hyperglycemia and examined eye development. The molecular mechanisms underlying this phenomenon were also studied. We identified hyperglycemia-mediated suppression of *Pax6* as a crucial target for its detrimental effects on chick embryo eye development. A decrease in *Pax6* gene expression induced by short hairpin RNA (shRNA) induced eye malformation in chick embryos. Overexpression of Pax6 could effectively rescue hyperglycemia-induced eye malformation. We demonstrated that O-linked *N*-acetylglucosaminylation (O-GlcNAcylation)-mediated oxidative damage is responsible for the suppression of the *Pax6* gene and hyperglycemia-induced eye malformations, which is consistent with the previous results.

## RESULTS

### Hyperglycemia increases eye abnormality in chick embryos

**Fig. 1.**
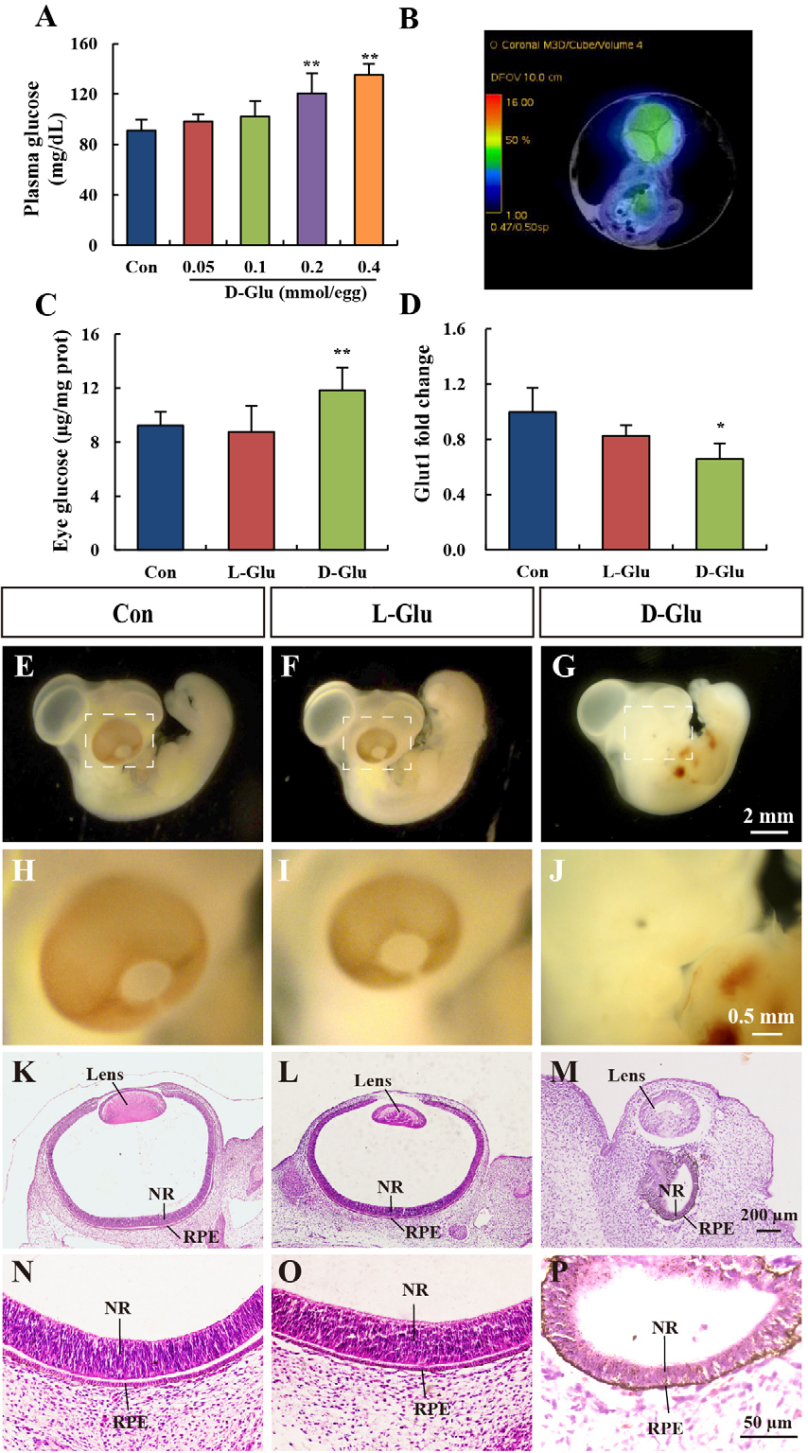
**D-glucose injection increased the glucose concentration in chick embryos and caused eye malformation.** (A) Plasma glucose concentration for EDD 5 embryos after air sac injection of varying concentrations of D-glucose at EDD 1. (B) PET-CT coronal image showing increased ^18^FDG uptake in the region of the eye. (C) The concentration of glucose in the eye resulting from injection of 0.2 mmol per egg L-glucose and D-glucose. (D) The gene expression of *Glut1* in the eye of EDD 5 control-, L-glucose-, and D-glucose-treated embryos. (E-P) Stereoscopic microscope measurement (E-J) and hematoxylin and eosin staining of embryo sections (K-P). Scale bars: 2 mm in E-G; 0.5 mm in H-J; 200 μm in K-M; 50 μm in N-P. Values were expressed as the mean±s.d. in each group (*n*=10). *P<0.05, **P<0.01 versus control. Con, control; D-Glu, treated with D-glucose; L-Glu, treated with L-glucose.

To induce hyperglycemia, different doses of glucose (0.05-0.4 mmol per egg) were injected into the air sac of chick embryos on EDD 1. The effects of exogenous glucose on embryonic development were detected on EDD 5. As shown in Fig. 1B, the doses of D-glucose at 0.2 and 0.4 mmol per egg increased the plasma glucose concentration of embryos significantly (Fig. 1A). As shown in Table 1, D-glucose at 0.2 mmol per egg significantly delayed embryonic development and increased the mortality and gross abnormality of chick embryos, especially eye abnormality. However, sham operations or vehicle controls had little influence on the eye development of chick embryos. L-glucose, an osmotic control, caused no significant delay in developmental stage when compared with control groups. Qualitative detection of glucose with PET imaging showed that chick embryos had obvious glucose enrichment around the region of the eyes (Fig. 1B). As shown in Fig. 1C, quantitative detection of glucose showed that D-glucose increased the eye glucose concentration significantly compared with vehicle or L-glucose control chick embryos. *Glut1*, a glucose transporter gene, was also measured. D-glucose treatment caused a significant reduction in the expression of *Glut1*, whereas L-glucose had no significant effect (Fig. 1D). Therefore, these results indicate that D-glucose increases eye glucose abnormalities in chick embryos. The morphology of embryos and transverse sections of eyes were also examined (Fig. 1E-P). Microphthalmos (Fig. 1G) was observed in chick embryos of the D-glucose-treated group. The size of the lens was decreased and it was translocated to the outside of the optic cup (Fig. 1M). In addition, the optic cup was closed and the retina was thinner (Fig. 1M,P).

**Table 1.**
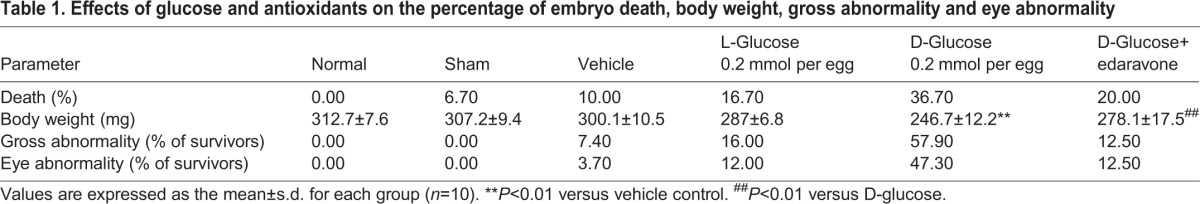
**Effects of glucose and antioxidants on the percentage of embryo death, body weight, gross abnormality and eye abnormality**

### Hyperglycemia impairs *Pax6* gene expression in early embryonic eyes

**Fig. 2.**
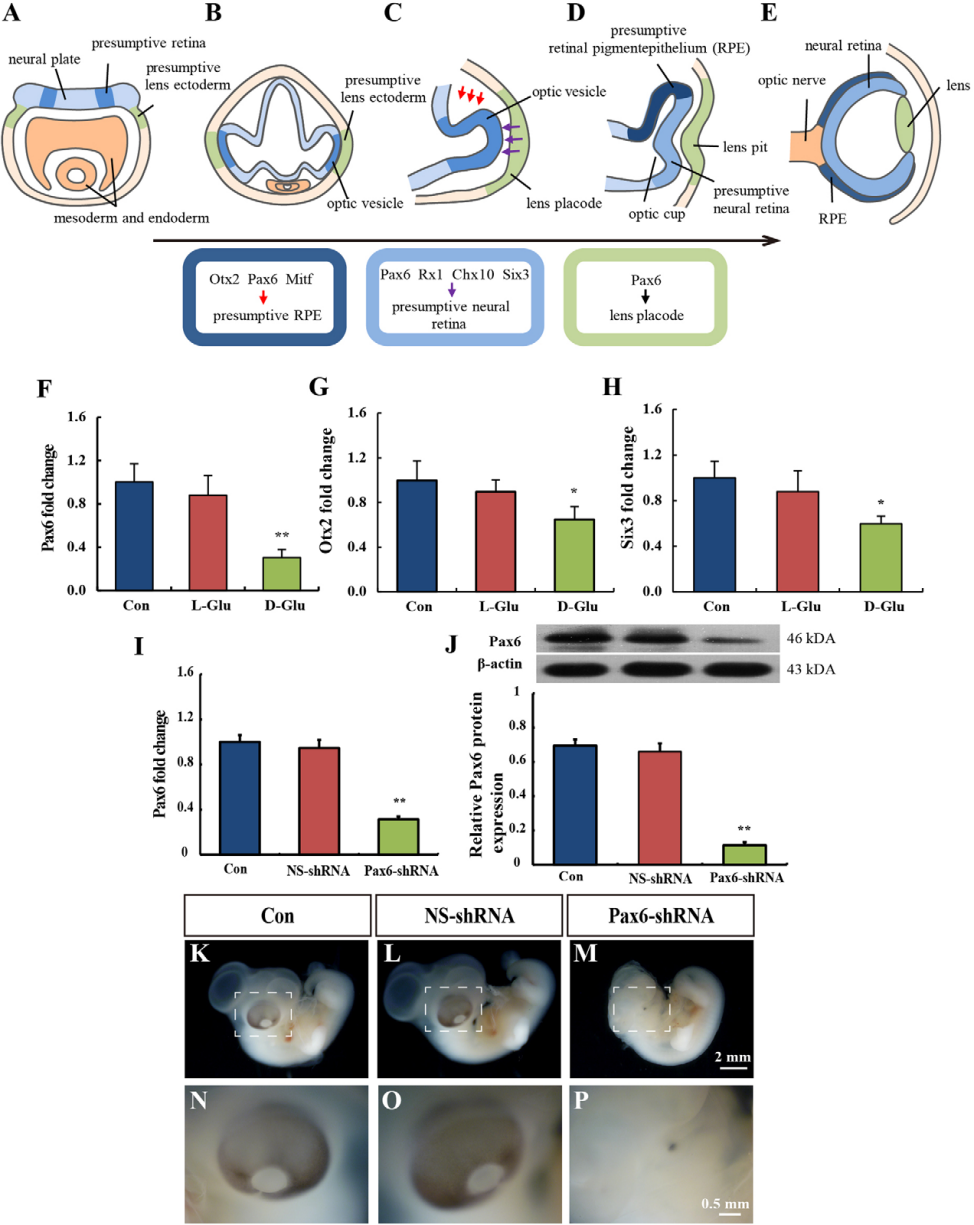
**Hyperglycemia impaired the gene expression of eye development markers.** (A-E) Schematic illustration of vertebrate eye development. (A) Transverse section of a neural plate-stage embryo. Orange indicates the mesoderm and endoderm. Light blue indicates the neural plate. Dark blue indicates the presumptive retina. The presumptive lens ectoderm is shown in light green. (B) Transverse section of a neural tube-stage embryo. The optic vesicle is developed from the presumptive retina field and reaches the presumptive lens ectoderm. (C-E) Lens and optic cup formation. The presumptive lens ectoderm becomes thickened to form the lens placode and subsequently invaginates from the ectoderm to form the lens vesicle and optic cup. At the bottom of A-E, expressions of presumptive retinal pigment epithelium genes, presumptive neural retina genes and lens placode genes (red, purple and black arrows, respectively) are indicated. (F-H) Hyperglycemia reduced the gene expressions of eye development markers *Pax6* (F), *Six3* (G) and *Otx2* (H). (I,J) *Pax6* gene (I) and Pax6 protein (J) levels were suppressed by *Pax6*-shRNA. (K-P) *Pax6*-shRNA caused eye malformation. Scale bars: 2 mm in K-M; 0.5 mm in N-P. Values were expressed as the mean±s.d. in each group (*n*=10). *P<0.05, **P<0.01 versus control. Con, control; NS, nonsilencing; RPE, retinal pigment epithelium.

Fig. 2A-E shows a schematic drawing that illustrates the vertebrate eye development program. This illustrates the relationship between eye development and the relative transcription factors. The molecular markers related to eye development in embryos on EDD 5 were examined. Results showed that the expression of *Pax6*, *Six3* and *Otx2* were significantly inhibited by hyperglycemia (Fig. 2F-H), but other genes, such as *Mitf*, *Rx1* and *Chx10*, were not affected significantly (data not shown). *Pax6* was the most vulnerable gene, being suppressed by 70% (Fig. 2F).

To examine whether *Pax6* expression has an important effect on the eye development, RNA interference (RNAi) was used to generate *Pax6* knockdown chick embryos. We found that microinjection of a control plasmid did not affect normal embryonic development (Fig. 2L,O). In chick embryos microinjected with a vector expressing shRNA against *Pax6*, the *Pax6* gene expression and Pax6 protein level were reduced by 60-80% (Fig. 2I,J). Microphthalmos was observed in *Pax6* knockdown chick embryos (Fig. 2M,P).

**Fig. 3.**
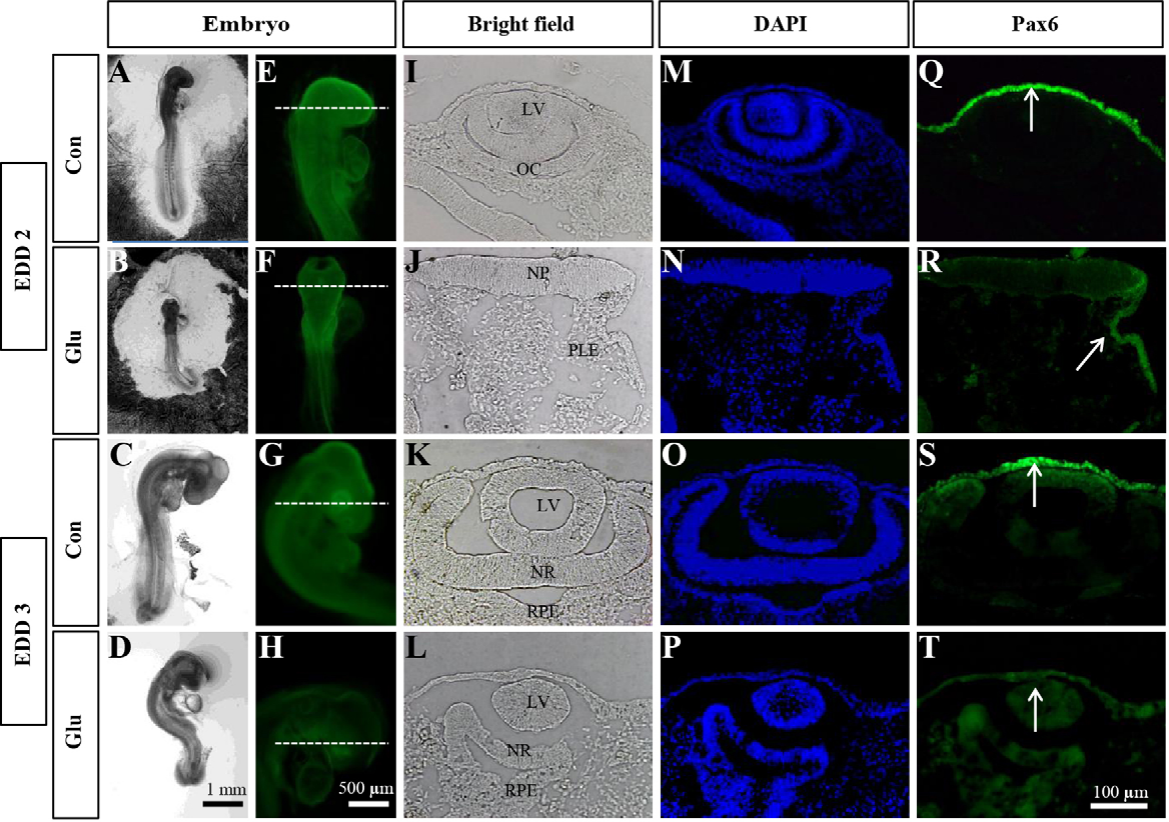
**Hyperglycemia impaired Pax6 protein expression in early embryo eyes.** Chick embryos were immunostained with Pax6 antibodies on EDD 2 and 3. (A-D) Bright-field images indicate the eye morphology. Scale bars: 1 mm. (E-H) Whole-mount immunostaining images show the expression of Pax6 (green stain). Scale bars: 500 μm. (I-T) Transverse sections (dotted white lines in E-H) were taken for bright-field images (I-L), DAPI (blue stain in M-P) and the expression pattern of Pax6 (green stain in Q-T). Scale bars: 100 μm. Con, control; Glu, treated with D-glucose; LV, lens vesicle; NP, neural plate; NR, neural retina; OC, optic cup; PLE, presumptive lens ectoderm; RPE, retinal pigment epithelium.

The spatiotemporal expression of the Pax6 protein was first detected in embryo eyes on EDD 2 and 3 by immunofluorescent staining (Fig. 3Q-T). At EDD 2, there was increased expression of Pax6 in the neuroectoderm and decreased expression in the optic cup, lens placode and retina (Fig. 3Q). In the D-glucose-treated embryos, the eye development was highly disorganized, and Pax6 could not be detected (Fig. 3R). In the control embryos at EDD 3, Pax6 was still abundantly expressed in the neuroectoderm and in the optic cup, lens and retina (Fig. 3S). In the corresponding D-glucose-treated embryos, the optic cup and the retinal anlagen were not well developed. The lens size was smaller than the control, and the expression of Pax6 was decreased (Fig. 3T).

### Plasmid pcDNA3.1(+)-*Pax6* rescues hyperglycemia-induced eye malformations

**Fig. 4.**
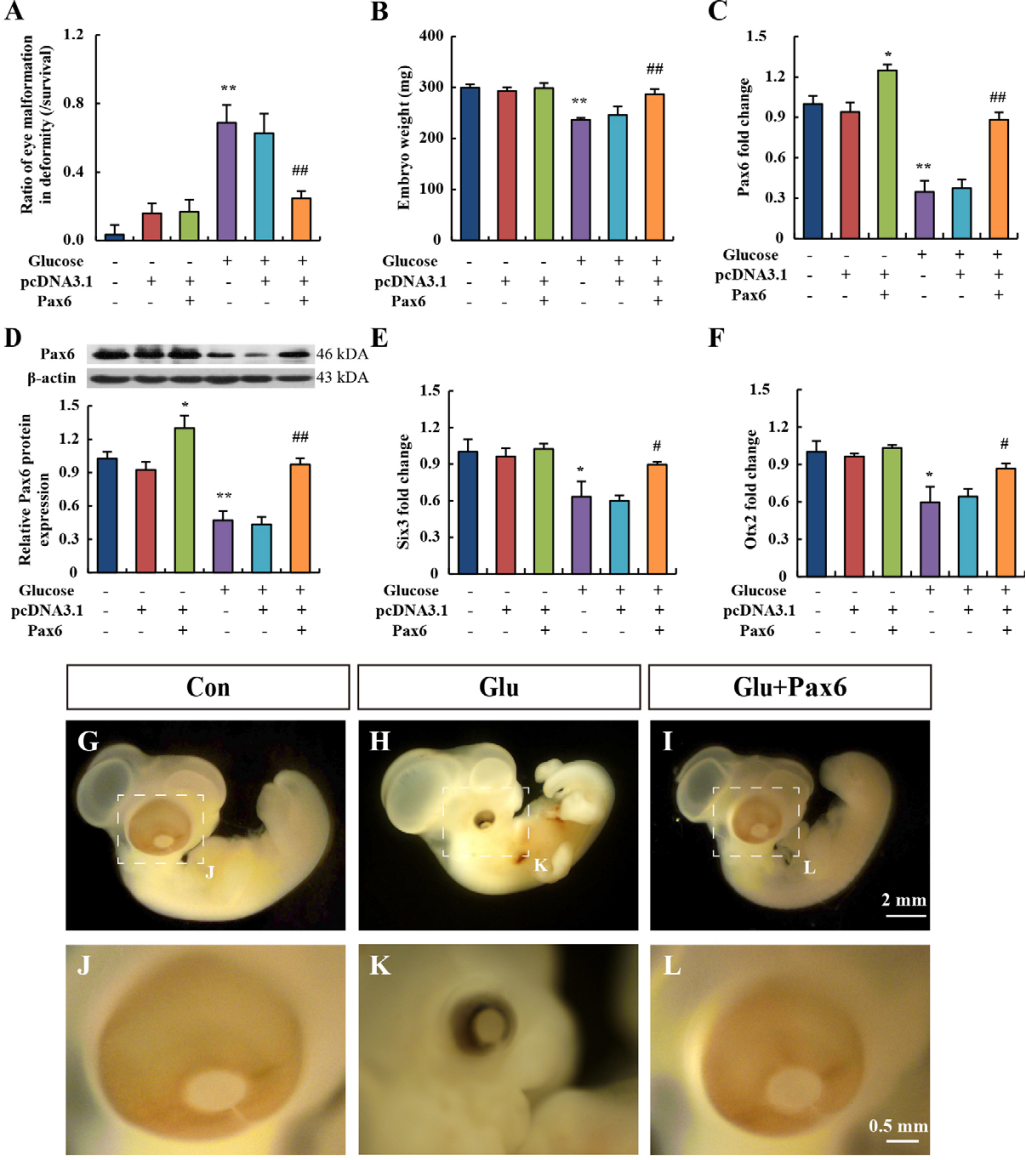
**Overexpression of *Pax6* rescued hyperglycemia-induced eye malformations.** Embryos on EDD 0 were injected with 1 μg pcDNA3.1(+)-*Pax6* plasmid, and then treated with 0.2 mmol per egg D-glucose on EDD 1. (A-F) Eye malformation ratio (A), body weight (B), *Pax6* gene expression (C) and Pax6 protein expression (D), *Six3* (E) and *Otx2* (F) gene expression of embryo eyes on EDD 5 were detected. Values were expressed as means±s.d. (*n*=10 in each group). *P<0.05, **P<0.01 versus control. ^#^*P*<0.05, ^##^*P*<0.01 versus glucose. (G-L) Stereoscopic microscope measurement of embryos. Scale bars: 2 mm in G-I; 0.5 mm in J-L. Con, control; Glu, treated with D-glucose.

To test whether hyperglycemia-mediated suppression of Pax6 is the crucial factor for eye malformations, a *Pax6* overexpression experiment was conducted in chick embryos. We found that microinjection of a vector plasmid (pcDNA3.1) and 1 μg of *Pax6* plasmid, respectively, did not affect normal embryonic development (Fig. 4A). The *Pax6* plasmid treatment increased the gene and protein expression of Pax6 in embryo eyes (Fig. 4C,D). *Pax6* plasmid promisingly restored high-glucose-induced eye malformations, dropping the malformation rate from 60 to 20% (Fig. 4A). This recovery was most distinguishable as *Pax6* prevented the occurrence of microphthalmos (Fig. 4I). The body weights of chick embryos were also increased by *Pax6* treatment (Fig. 4B). Moreover, the *Pax6* plasmid restored the expression of *Six3* and *Otx2* to levels that were nearly normal (Fig. 4E,F), suggesting that hyperglycemia-induced suppression of *Pax6* is the upstream event.

### *Pax6* suppression is caused by hyperglycemia-induced oxidative stress in chick embryos

**Fig. 5.**
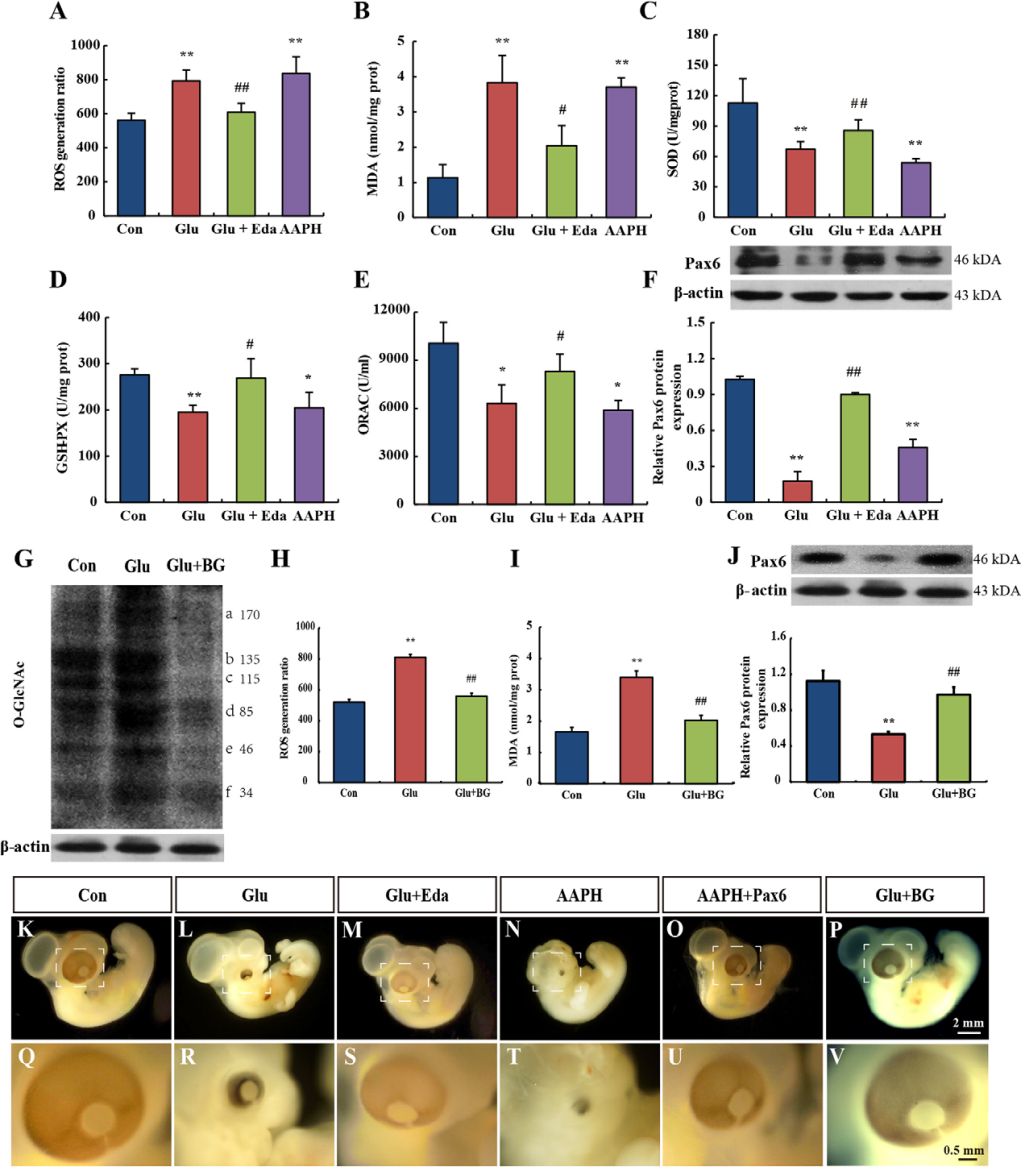
**Oxidative stress induced by hyperglycemia in chick embryos.** (A-F) ROS generation ratio (A), MDA level (B), SOD (C) and GSH-PX (D) activities, ORAC level (E) and Pax6 protein expression (F) were measured in the eyes of EDD 5 embryos for the control-treated, D-glucose-treated, D-glucose- plus edaravone-treated and AAPH-treated embryos. (G) Hyperglycemia caused significantly elevated O-GlcNAcylation at the protein level. (H,I) Hyperglycemia-induced oxidative stress was alleviated by O-GlcNAcylation inhibitor BG. (J) The decreased Pax6 protein was restored after supplementation of BG. Values were expressed as means±s.d. (*n*=10 in each group). *P<0.05, **P<0.01 versus control. ^#^*P*<0.05, ^##^*P*<0.01 versus glucose. (K-V) Stereoscopic microscope measurement of embryos. Scale bars: 2 mm in K-P; 0.5 mm in Q-V. AAPH, 2,2′-azobis(2-amidinopropane) dihydrochloride; BG, benzyl-2-acetamido-2-deoxy-α-D-galactopyranoside; Con, control; Eda, edaravone; Glu, treated with D-glucose.

We also investigated the level of reactive oxygen species (ROS) production and antioxidative ability in the eyes of chick embryos. As shown in Fig. 5A, ROS generation was significantly increased in the eyes of chick embryos on EDD 5 after D-glucose treatment. Similar results were observed as an increase in malondialdehyde (MDA) content (Fig. 5B). The activities of total superoxide dismutase (SOD) and glutathione peroxidase (GSH-PX) were decreased in the D-glucose-treated eyes (Fig. 5C,D). Also, the oxygen radical absorbance capacity (ORAC) level was lower in D-glucose-treated chick embryo eyes than that in vehicle control (Fig. 5E). However, edaravone, a well-known antioxidant, could effectively alleviate malformations induced by D-glucose (Fig. 5M,S). Oxidative stress markers are shown in Fig. 5A-E. Edaravone prevented weight loss compared with the glucose group (Table 1). Edaravone also greatly restored Pax6 expression (Fig. 5F).

To verify the causality between oxidative stress and hyperglycemia-induced eye malformation, we treated embryos with 2,2′-azobis(2-amidinopropane) dihydrochloride (AAPH), a free radical generator. Similar to the effect of D-glucose, AAPH also caused microphthalmos (Fig. 5N,T). Consistently, Pax6 protein expression in eyes was significantly decreased (Fig. 5F). However, *Pax6* plasmid microinjection could prevent the occurrence of microphthalmos (Fig. 5O,U).

We also found that the overall O-GlcNAc level of the eye was increased in the hyperglycemic conditions (Fig. 5G). A similar trend was observed for O-GlcNAc levels on individual protein bands (Fig. 5G, bands a-f). To determine the role of O-GlcNAcylation in this model, we administered the inhibitor of O-GlcNAcylation, benzyl-2-acetamido-2-deoxy-α-D-galactopyranoside (BG). BG prevented hyperglycemia-induced ROS and MDA formation (Fig. 5H,I) and significantly restored Pax6 protein expression (Fig. 5J). The embryo morphology showed that BG prevented eye malformations (Fig. 5P,V). Therefore, we speculated that hyperglycemia-induced oxidative stress was mediated by O-GlcNAcylation. Oxidative stress-mediated *Pax6* suppression plays a pivotal role in hyperglycemia-induced eye malformation.

## DISCUSSION

Epidemiological surveys have shown that children born to a diabetic mother have a higher risk of having eye malformations (Tariq et al., 2010). However, few models have been used to study the underlying mechanisms of the congenital eye malformations. In the present study, we used chick embryos to establish a new model to study the direct effect of high glucose on embryonic eye development. Chick embryos previously exposed to 0.2 mmol per egg D-glucose had a sustained elevation in plasma and eye glucose content until EDD 5. Positron emission computed tomography (PET-CT) results demonstrated that the distribution of glucose was mainly in the regions of the eye and brain in chick embryos. This supported the results showing that the injection of glucose elevated the glucose concentration in the eye of chick embryos. Hyperglycemic conditions can induce osmotic stress through increased activity in the polyol pathway and excess generation of sorbitol, which alters membrane permeability and causes cell lesions (Oishi et al., 2002). Thus, we used L-glucose as an osmotic control because glucose transporters only facilitate the transportation of D-glucose across the cell membrane. As predicted, L-glucose did not elevate the eye glucose concentration as much as did D-glucose. Glut1 mediates the cellular uptake of glucose into tissues. It is expressed in the endothelial and epithelial barriers, including the eyes (Kumagai et al., 1994). In the present study, sustained hyperglycemic conditions resulted in a decrease in the gene expression of *Glut1* in the eyes of chick embryos. This could be explained as a compensatory mechanism in response to a high glucose concentration in the eye. D-glucose-induced elevation of blood glucose also reduced the weights of the embryos and induced eye malformations. Specifically, hyperglycemia affected the development of the retina and lens. Microphthalmos was observed in 50% of the embryos (Table 1).

In order to understand the underlying mechanisms, several molecular markers of eye development, including *Pax6* (Shaham et al., 2013), *Six3* (Zhu et al., 2002) and *Otx2* (Rath et al., 2007), were studied. The results showed that all three genes were downregulated after D-glucose treatment. Among these genes, *Pax6* was the most sensitive to glucose fluctuation in embryos. *Pax6* is a master transcriptional gene that regulates the formation of the optic vesicle, optic cup, lens placode and retina (Gehring, 2005; Shaham et al., 2013; Walther and Gruss, 1991). Previous research had suggested that *Pax6* mutations could lead to human aniridia, anterior segment dysgenesis and microphthalmia (Glaser et al., 1992; Hanson et al., 1994; Xiao et al., 2012). These studies clearly demonstrated the importance of *Pax6* in eye development. In our present study, suppression of *Pax6* expression could induce eye malformation in chick embryos. Interestingly, we found that Pax6 was expressed strongly in the neuroectoderm, but weakly in the optic cup, lens placode and retina on EDD 2. After 24 h of development, Pax6 expression was gradually enhanced in the optic cup, lens and retina, which indicated that eye morphogenesis was possibly a consequence of Pax6 protein migration. As previously shown, conditional deletion of *Pax6* in the placode prevented placodal thickening, lens pit invagination and optic cup morphogenesis (Smith et al., 2009). In our study, overexpression of *Pax6* rescued the hyperglycemia-induced eye malformation. The restoration of *Pax6* also improved the expression of *Six3* and *Otx2.* Six3 and Otx2 could possibly be the downstream regulators of Pax6 in eye development. These results suggested that hyperglycemia impaired Pax6 expression, which caused eye malformation.

O-GlcNAcylation is one of the post-translational modifications that modify transcription factors and is involved in the translation and degradation process of proteins. It is a nutritionally responsive modification (Hart, 1997). Dysregulation of O-GlcNAcylation is implicated in the pathogenesis of diabetes (Buse, 2006). An elevated O-GlcNAc level was found in the total protein of chick embryos after high glucose exposure. O-GlcNAc is maintained by two highly conserved enzymes, O-GlcNAc transferase (OGT) and O-GlcNAcase (OGA; Lima et al., 2012). The donor substrate for OGT activity, UDP-GlcNAc, is a terminal product of the hexosamine biosynthesis pathway (HBP). Flux through the HBP and UDP-GlcNAc levels change rapidly according to nutrient conditions (Hanover et al., 2010). The activation of HBP can induce ROS generation or oxidative stress, which is associated with hyperglycemia (Joseph et al., 2014). Goldberg et al. (2011) found that O-GlcNAc depletion in mesangial cells could prevent high-glucose-induced ROS formation. In our present study, BG, the inhibitor of O-GlcNAcylation, prevented hyperglycemia-induced ROS and MDA formation. These results indicated that hyperglycemia-induced oxidative stress might be related to O-GlcNAcylation.

It has been reported that hyperglycemia could promote ROS production, whereas preventing ROS production could compensate for the effects of hyperglycemia (Sethi et al., 2006). During embryogenesis, insulin is not yet produced by the conceptus and could not be obtained from the maternal circulation (Loeken, 2006). Pancreatic insulin does not appear in chick embryos until EDD 3.5-4 (Dieterlen-Lievre and Beaupain, 1976). Disturbances in metabolism during early pregnancy are responsible for defective organogenesis in diabetic pregnancies (Naftolin et al., 1987). The embryo has a high level of oxygen consumption and is exceptionally vulnerable to oxidative damage, but its antioxidant defenses are not well developed. The elevation of ROS during oxidative stress has long been linked to diabetes or GDM (Jin et al., 2013; Newsholme et al., 2010; Nishikawa and Araki, 2007). ROS are thought to exert their deleterious effects primarily by damaging virtually all classes of biomolecules, including DNA, proteins and lipids (Bitar and Al-Mulla, 2012), leading to cell death. It has been proposed that oxidative damage contributes to the development of diabetic retinopathy (Kowluru et al., 2001). In addition, in chick embryos, hyperglycemia reduced embryo viability (Miller et al., 2005). In this study, we have found that the eye malformation in the chick embryos was probably the result of an excess of ROS generation after D-glucose treatment. In order to prove this hypothesis, we used a peroxyl radical generator, AAPH, to mimic the hyperglycemia-induced oxidative stress status. AAPH could induce eye malformation and impair *Pax6* expression in a similar manner to D-glucose treatment. Promisingly, the use of the antioxidant edaravone prevented eye malformation. Pax6 protein expression was also increased. These phenomena proved that the decreased expression of *Pax6* caused by oxidative stress was the main reason for hyperglycemia-induced eye malformation. Consistently with our study, it was demonstrated that Pax6 was susceptible to oxidative stress (Ou et al., 2008). Pax6 protein could easily be oxidized and excluded from the nucleus of stressed corneal epithelial cells, with concomitant loss of corneal epithelial markers. It is therefore conceivable that high glucose might induce eye malformation in chick embryos by increasing the oxidative stress status within the eye, which in turn suppresses the expression of Pax6.

In summary, we successfully developed a new GDM model using the chick embryo to study the molecular mechanism of hyperglycemia-induced eye malformation. We also showed that high glucose induced oxidative stress status, where Pax6 is a crucial target. However, the effects of GDM on the developing embryo are complex. Much work still needs to be done in order to reach a complete understanding of this phenomenon. There are limitations to the chick model. For example, the non-maternal environment of the chick embryo cannot imitate the human situation. Despite this, we believe that the chick embryo model has its own advantages for studying GDM-related diseases, including well-defined developmental stages, the rapid rate of development and ease of manipulation.

## MATERIALS AND METHODS

### Animals

Fertilized leghorn eggs were purchased from Avian Farm of South China Agriculture University (Guangzhou, China). The eggs were incubated in a humidified incubator (Grumbach, Wetzlar, Germany) at 38°C and 70-75% relative humidity. Different concentrations of D-glucose (Sigma-Aldrich, St Louis, MO, USA) at 0.05, 0.1, 0.2 and 0.4 mmol per egg, L-glucose (0.4 mmol per egg, osmotic control; Sigma-Aldrich) or chick saline (vehicle control, 0.72%) were injected into the air sac of the embryos on EDD 1. The embryos were sampled and weighed on EDD 5. The mortality was measured by counting the number of dead versus total embryos. Gross malformation in the embryos was assessed as the number of gross abnormal versus surviving embryos. Eye abnormality was assessed as abnormal eye development versus surviving embryos. For the oxidative stress experiment, chick embryos were treated with 0.2 mmol per egg glucose plus 0.1 nmol per egg edaravone and AAPH (5 μmol per egg; Sigma-Aldrich) on EDD 1. The O-GlcNAcylation inhibitor, benzyl-2-acetamido-2-deoxy-α-D-galactopyranoside (BG; 1 µmol per egg; Santa Cruz Biotechnology, Santa Cruz, CA, USA) was administered with glucose at EDD 1. After treatment, all eggs were incubated for a further 1, 2 or 3 days. There were 30 eggs in each group. All experiments using chick embryos were performed according to the guidelines of the Jinan University Institutional Animal Care and Use Committee.

### Measurement of plasma and eye glucose concentrations

Whole blood was collected from chick embryos using a modified glass capillary syringe under a microscope at EDD 5. Eyes were isolated from individual embryos and homogenized with cold avian saline to obtain a 20% homogenate. The heparin-containing blood and the homogenate were centrifuged at 1000 ***g*** for 10 min to obtain the supernatant. Glucose concentrations in the plasma and eyes were measured using a glucose oxidase-coupled spectrophotometric assay kit (Sigma-Aldrich) according to the manufacturer's instructions.

### Positron emission computed tomography scanning

In order to investigate the distribution of glucose in different organs, chick embryos with complete organ development at EDD 15 (Hamburger and Hamilton, 1992) were used. A warm (37.5°C) tracer solution containing 1.0 μCi of 2-deoxy-2-[^18^F]fluoro-D-glucose (^18^FDG) was promptly but gently deposited directly onto the region of the intact shell membrane. After 30 min, PET images were obtained for evaluation. As a result of the high physiological clearance rate of glucose and the half-life of ^18^F, the vast majority of ^18^FDG had been cleared from the bloodstream in 30 min. Further cellular incorporation of any remaining glucose would have little effect on PET image intensities. The PET images therefore represent glucose uptake by cells during the recording period.

### Histological analysis

Eye damage was assessed by histological examination of sections from embryos on EDD 5. The embryos were immersed in 4% paraformaldehyde for 3 days before paraffin embedding. The paraffin sections were sliced at 5 μm thickness and were processed for hematoxylin and eosin staining.

### Immunofluorescent staining

Immunofluorescent staining was performed to detect Pax6 expression on EDD 2 and EDD 3. The embryos were incubated overnight at 4°C on a rocker with primary monoclonal antibody mixtures raised against Pax6 (1:100; Developmental Studies Hybridoma Bank, University of Iowa, IA, USA; catalog no. AB 528427). After an extensive wash with PBS, the embryos were incubated overnight at 4°C on a rocker once again with specific secondary antibody mixtures coupled with Alexa Fluor 488 (1:1000; Invitrogen, Carlsbad, CA, USA; catalog no. CA11001s) to visualize the primary antibodies. The embryos were photographed under a fluorescence stereomicroscope (Olympus MVX10, Hamburg, Germany) and then embedded in optimum cutting temperature compound for frozen sections (16 μm thick; Leica CM 1900). Transverse sections of the embryos were photographed under a fluorescence microscope (Olympus IX51, Hamburg, Germany) and a confocal microscope (Zeiss LSM 700, Jena, Germany).

### Real-time PCR

For quantitative real-time PCR analysis, the total RNA of embryo eyes on EDD 5 was extracted using TRIzol reagent (Takara, Kyoto, Japan) according to the manufacturer's protocol. Total RNA (3 μg) was reverse transcribed into cDNA at 42°C for 1 h in 20 μl of reaction mixture containing reverse transcriptase with oligo(dT)15 primer (Tiangen, Beijing, China). The cDNA was then determined using Maxima™ SYBR Green/Fluorescein qPCR Master Mix (Fermentas, Hanover, MD, USA) via the IQTM5 real-time PCR detection system (Bio-Rad, Hercules, CA, USA). The final products were analyzed by the cycle threshold method. The forward and reverse primers, respectively, for *Pax6*, *Glut1*, *Six3*, *Otx2* and *β-actin* were as follows: *Pax6*, 5′-GCTATGACACCTACAC-3′ and 5′-ACTTGAACTGGAACTG-3′; *Glut1*, 5′-TCTCTGTCGCCATCTTCTCG-3′ and 5′-TGGTGAGGCCAGAATACAGG-3′; *Six3*, 5′-CCAGTGTTTCCAGTTTGA-3′ and 5′-TTGTTGTTGTTGTTGTGATT-3′; *Otx2*, 5′-ACCTCAACCAGTCTCC-3′ and 5′-TCCAAGCAGTCAGCAT-3′; and *β-actin*, 5′-TACCTTCAACTCCATCA-3′ and 5′-CTCCAATCCAGACAGA-3′.

### Plasmid construction and microinjection

RNAi candidate target sequences were designed based on the chick *Pax6* mRNA sequence and cloned into the pGMLV-SCS vector (Integrated Biotech Solutions, Shanghai, China). The RNAi sequence GGGAGAACACCAACTCCATCA was used in experiments to knock down endogenous *Pax6*. Nonsilencing (NS) small interfering RNA (shRNA) was also cloned into the pGMLV-SCS vector (Integrated Biotech Solutions). For gain-of-function experiments, the coding sequence of chick *Pax6* (NM_205066) was chemically synthesized by the Invitrogen company, and then subjected to PCR amplification with the following primers: *Pax6*-F, 5′-AA*GGATCC*GCCACCATGCAGAACAGTCACAGC-3′ (the *Bam*HI site is shown in italic, and the Kozak sequence is underlined) and *Pax6*-R, 5′-CG*GAATTC*TTACTGTAATCTTGGCCAATACTG-3′ (the *Eco*RI site is shown in italic). The resulting PCR products were treated with *Eco*RI and *Bam*HI and inserted into *Eco*RI/*Bam*HI-treated pcDNA3.1(+) to give plasmid pcDNA3.1(+)-*Pax6*. Five microliters of *in vivo*-jetPEI^®^ (Polyplus-transfection, Illkirch, France; catalog no. PO 101-10N) containing concentrated plasmid (1 μg) was injected into the central part of the subgerminal cavity before incubation using a microinjection pipette according to the manufacturer's instructions (Polyplus-transfection, New York, NY, USA). Glucose was injected as described previously. The embryos were incubated to EDD 5 to determine eye development.

### Measurement of MDA contents, SOD and GSH-PX activities, ORAC level and ROS generation ratio in chick embryo eyes

Peroxide content in the embryo eyes on EDD 5 was determined by a commercial MDA kit (Nanjing Jiancheng Institute of Biotechnology, Nanjing, China). The activities of total SOD and GSH-PX were measured using commercial kits according to the manufacturer's instructions (Nanjing Jiancheng Institute of Biotechnology). ROS generation was detected with 5 μM of 2′,7′-dichlorofluorescein-diacetate (DCFH-DA; Sigma-Aldrich). The ORAC procedure was modified from the previously described method (Kurihara et al., 2004).

### Western blotting analysis

Embryo eyes were separated and lysed in lysis buffer (Beyotime Institute of Biotechnology, Shanghai, China). Proteins (30 μg) were separated by SDS-PAGE and blotted onto nitrocellulose membranes (Amersham Biosciences, Piscataway, NJ, USA). The membranes were individually incubated with anti-Pax6 (1:1000; Developmental Studies Hybridoma Bank, University of Iowa, IA, USA; catalog no. AB 528427), anti-O-GlcNAc (1:2000; Sigma-Aldrich; catalog no. O7764) and anti-β-actin (1:2000; Developmental Studies Hybridoma Bank; catalog no. 224-236-1). Subsequently, the membranes were incubated with goat anti-rabbit or goat anti-mouse IgG secondary antibody (Cell Signaling Technology, Danvers, MA, USA). The immunodetection was done using an enhanced chemiluminescence detection kit (MultiSciences Biotech Co., Ltd, Beijing, China). The band density was quantified using Quantity One analysis software (Bio-Rad).

### Statistical analysis

Experimental values are given as means±s.d. One-way analysis of variance (ANOVA) was used to investigate differences in the data of biochemical parameters among the different groups, followed by Dunnett's significant *post hoc* test for pairwise multiple comparisons. Differences were considered as statistically significant at *P*<0.05.
